# Identification and annotation of newly conserved microRNAs and their targets in wheat (*Triticum aestivum* L.)

**DOI:** 10.1371/journal.pone.0200033

**Published:** 2018-07-10

**Authors:** Habibullah Khan Achakzai, Muhammad Younas Khan Barozai, Muhammad Din, Iftekhar Ahmed Baloch, Abdul Kabir Khan Achakzai

**Affiliations:** Department of Botany, University of Balochistan, Quetta, Balochistan, Pakistan; Nigde Omer Halisdemir University, TURKEY

## Abstract

MicroRNAs (miRNAs) are small, non-coding and regulatory RNAs produce by cell endogenously. They are 18–26 nucleotides in length and play important roles at the post-transcriptional stage of gene regulation. Evolutionarily, miRNAs are conserved and their conservation plays an important role in the prediction of new miRNAs in different plants. Wheat (*Triticum aestivum* L.) is an important diet and consumed as second major crop in the world. This significant cereal crop was focused here through comparative genomics-based approach to identify new conserved miRNAs and their targeted genes. This resulted into a total of 212 new conserved precursor miRNAs (pre-miRNAs) belonging to 185 miRNA families. These newly profiled wheat’s miRNAs are also annotated for stem-loop secondary structures, length distribution, organ of expression, sense/antisense orientation and characterization from their expressed sequence tags (ESTs). Moreover, fifteen miRNAs along with housekeeping gene were randomly selected and subjected to RT-PCR expressional validation. A total of 32927 targets are also predicted and annotated for these newly profiled wheat miRNAs. These targets are found to involve in 50 gene ontology (GO) enrichment terms and significant processes. Some of the significant targets are RNA-dependent DNA replication (GO:0006278), RNA binding (GO:0003723), nucleic acid binding (GO:0003676), DNA-directed RNA polymerase activity (GO:0003899), magnesium ion transmembrane transporter activity (GO:0015095), antiporter activity (GO:0015297), solute:hydrogen antiporter activity (GO:0015299), protein kinase activity (GO:0004672), ATP binding (GO:0005524), regulation of Rab GTPase activity (GO:0032313) Rab GTPase activator activity (GO:0005097), regulation of signal transduction (GO:0009966) and phosphoprotein phosphatase inhibitor activity (GO:0004864). These findings will be helpful to manage this economically important grain plant for desirable traits through miRNAs regulation.

## 1. Introduction

MicroRNAs (miRNAs) are a special abundant regulatory class of RNAs known for properties such as non-coding, endogenous in nature and short lengths from 18 to 26 nucleotide (nt). These small miRNAs are called as mature miRNAs, generate from long precursor miRNAs (pre-miRNAs) whose length ranges from 70–500 nt and forming a self-folded stem-loop secondary structures by Dicer-like 1 (DCL1) enzyme in plants [1]. Mature miRNAs are involved to regulate gene expression at post-transcriptional levels by either targeting mRNAs for degradation or hindering protein translation. Both processes are accomplished by the complementary base pairing of miRNAs to their target mRNA sequences [2]. In plants, for a majority of cases, miRNAs interact with their targets through perfect or near-perfect hybridizing and lead to target mRNA degradation [3]. Growing confirmation has revealed that miRNAs play a significant role in an extensive range of developmental processes in plants, including cell proliferation, stress response, metabolism, inflammation, and signal transduction [2–4]. To date, more than 28,645 miRNAs have been reported from 223 species of plants and animals and available in the publicly available database miRBase (Release 21) [5]. The majority of plant miRNAs have been identified in species with fully sequenced genomes such as; 713 from *Oryza sativa*, 401 from *Populus trichocarpa*, 384 from *Arabidopsis thaliana*, 343 from *Solanum tuberosum*, 321 from *Zea mays*, and 241 from *Sorghum bicolor* [5]. miRNA-related research is constantly increasing and miRNAs, along with their functions, are being profiled and annotated through various computational tools and experimental methods such as direct cloning, deep sequencing, and other approaches. Comparison of miRNAs through several plant species has shown that some miRNAs are greatly evolutionary conserved from species to species, such as from mosses to higher flowering eudicots in the plant kingdom [4]. Conservation nature of miRNAs has provided a valid approach for profiling new miRNAs in other species. Presently, comparative genome-based approaches have been used to profile conserved miRNAs in many plant species, such as cotton [6], switchgrass [7, 8], soybean [9], tomato [10], chilli [11], roses [12], helianthus [13], cherry [14], red alga [15] cowpea [16]. Wheat (*T*. *aestivum* L.) is belongs to family Poaceae and also known as bread wheat. Wheat, based on production, is the world second most-produced cereal crop after maize [17]. It is an important source of carbohydrates, vegetal protein and multiple nutrients and dietary fiber [18]. In the miRBase, a database of miRNAs (http://www.mirbase.org/, Release 21: June 2014) [5], only 119 mature miRNAs are reported in this significant staple food. Although progress has been made on the identification and profiling of miRNAs in the wheat [19–21], still a need to profile more conserved miRNAs is fruitful for this significant cereal crop. In this study, a well-defined comparative genome based homolog search was employed to profile new wheat miRNAs and their targets.

## 2. Materials and methods

### 2.1. Obtaining reference miRNA sequences

A total of 6396 plant precursor and mature miRNA sequences were obtained from the miRBase, a database of miRNAs (http://www.mirbase.org/, Release 21: June 2014) [5]. These reference miRNAs were belonged to 26 plant species, as *Oryza sativa* (osa), *Brachypodium distachyon* (bdi), *Hordeum vulgare* (hvu), *Sorghum bicolor* (sbi), *Glycine max* (gma), *Medicago truncatula* (mtr), *Populus trichocarpa* (ptc), *Prunus persica* (ppe), *Arabidopsis thaliana* (ath), *Vitis vinifera* (vvi), *Brassica rapa* (bra), *Gossypium raimondii* (gra), *Solanum tuberosum* (stu), *Citrus sinensis* (csi), *Gossypium hirsutum* (ghr), *Cynara cardunculus* (cca), *Carica papaya* (cpa), *Solanum lycopersicum* (sly), *Brassica napus* (bna), *Arachis hypogaea* (ahy), *Acacia auriculiformis* (aau), *Amborella trichopoda* (atr), *Populus euphratica* (peu), *Nicotiana tabacum* (nta), *Hevea brasiliensis* (hbr). The 6396 miRNAs were used as the reference miRNAs to predict new conserved miRNAs from the wheat expressed sequences tags (ESTs).

### 2.2. Retrieval of candidate miRNAs

To mine new conserved wheat miRNAs through comparative homology-based search, a total of 1,286,372 wheat ESTs were downloaded from the EST-database (dbEST), (release 130101, 1 January 2013) available at https://www.ncbi.nlm.nih.gov/genbank/dbest/dbest_summary. The reference miRNAs and wheat ESTs were subjected to BLASTn and BLASTx algorithms to profile potential conserved miRNAs and remove repeated sequences and protein coding sequences respectively [22]. The FASTA format of potential candidate wheat miRNAs, having maximum four mismatches with the reference miRNAs and non-coding in nature were selected, saved and subjected to downstream analyses.

### 2.3. Prediction of wheat miRNAs secondary structures

Prediction of stem-loop secondary structures of initial potential candidate sequences is an important criterion for profiling and characterization of new conserved miRNAs in wheat [2]. MFOLD (version 3.6) [23], a secondary structure prediction tool was employed to produce stem-loop structures for the initial identified potential wheat miRNA sequences. All the initial candidate sequences who failed to develop stable secondary structures were discarded. Only potential candidate miRNA sequences with stable stem-loop structures showing mature sequences in the stem region, at least 12 nucleotide involved in Watson-Crick or G/U base pairing with the opposite strand and having minimum free energy (MFE) ≤ -10Kcalmol^-1^ were saved and subjected to physical scrutiny.

### 2.4. Physical scrutiny

Physical scrutiny of the candidate miRNAs is an essential step to exclude the false positive miRNAs. So, all the potential candidate miRNAs resulted from the wheat ESTs with properties such as having maximum 4 mismatches with the reference miRNAs, non-coding in natures, forming a stable stem-loop secondary structure and single-tone in natures were subjected to physical scrutinization to remove the sequences with large bulges, mature sequences not in the stem-region and having higher MFEs. The organ of expression for each of the newly profiled wheat miRNA is also noted from its EST.

### 2.5. RT-PCR validation

From the newly profiled wheat miRNAs, fifteen miRNAs were randomly selected and subjected to expressional analysis by RT-PCR (Reverse Transcription) along with Ta54227 (Cell division control prot., AAA-superfamily of ATPases), as an housekeeping gene [24]. Primer-3 algorithm (http://bioinfo.ut.ee/primer3-0.4.0) was used to design stem-loop primers (S1 Table) from the ESTs of 15 randomly selected miRNAs. Total RNA was extracted from the leaves of wheat using Qiagen plant RNA kit. Later, cDNA was synthesized using the RevertAid™ First Strand cDNA synthesis Kit (Fermentas), according to the supplier's protocol. A 60 μg cDNA was used as template and PCR was programmed as follows: initial denaturation at 95°C for 3 min, for 30 cycles; denaturation at 94°C for 35 sec, annealing at 60°C for 35 sec, and extension at 72°C for 30 sec and final elongation step at 72°C for 10 min. The PCR products were separated through 1.5% (w/v) agarose gel with 100 base pair DNA leader.

### 2.6. Targets prediction

In order to predict putative targets for the newly profiled wheat miRNAs, psRNATarget, a plant small RNA target analysis server available at http://plantgrn.noble.org/psRNATarget/ [25] was used. The wheat library (*Triticum aestivum* (wheat), cDNA, EnsemblPlants, release-31) was used as selected target library with the modified 2017-updated parameters of psRNATarget as Max Expectation cutoff: 5, HSP length for scoring: 19, Penalty for GU pair: 0.5, Penalty for other mismatch: 1.0, Allowing bulge on target: Yes, Penalty for opening gap: 2.0, Penalty for extending gap: 0.5, Weight for seed region: 1.5, Seed region: 2–13, # of mismatches allowed in seed region: 2 and Calculating UPE: No. The predicted putative wheat miRNA targets were subjected to the Gene Ontology functional and enrichment analyses through agriGO [26].

## 3. Results and discussion

### 3.1. New potential miRNAs in wheat

Comparative genomics based research is a well-known approach for new interesting findings in various organisms [27–31]. Here, the comparative genomics-based homology search produced a total of 212 new conserved miRNAs from wheat ESTs (S2 Table). The 212 new conserved miRNAs are belonged to 185 miRNA families. These are tae-miR413, 435, 476, 477a, 477b, 529, 815, 818a, 818b, 827, 854, 858, 1428, 1432, 1435a, 1435b, 1435c, 1436, 1437, 1438, 1439, 1444, 1445, 1516, 1522, 1535, 1848, 1858, 1861a, 1861b, 1866, 1869, 1878, 1882, 1913, 2086, 2088a, 2088b, 2094, 2104, 2106, 2118a, 2118b, 2118c, 2118d, 2122, 2636, 2643, 2905, 2924, 2926, 2927, 3476, 3513, 3522, 3627, 3633, 3635, 3636, 3954, 4364, 4367, 4411, 4993, 4995, 5017, 5034, 5039, 5040, 5056, 5059, 5063, 5064, 5070, 5075, 5076, 5082, 5083, 5161, 5167a, 5167b, 5169a, 5169b, 5171, 5174a, 5174b, 5183, 5203, 5225, 5233, 5234, 5254, 5265, 5272, 5288, 5291, 5298, 5338, 5386, 5387, 5490, 5502, 5508, 5523, 5527, 5538, 5539, 5543, 5562, 5564, 5565a, 5565b, 5565c, 5565d, 5565e, 5565f, 5565g, 5565h, 5568a, 5568b, 5568c, 5568d, 5568e, 5641, 5660, 5679, 5721, 5783, 5806, 5809, 5814, 5824, 5833, 6026, 6034, 6035, 6111, 6116, 6164, 6177, 6179, 6180, 6181, 6182, 6183, 6184, 6187, 6188, 6189, 6190, 6191a, 6191b, 6192, 6193, 6195, 6196, 6199, 6202, 6204, 6205, 6207, 6209, 6213, 6214, 6220, 6224a, 6224b, 6225, 6233, 6246, 6248, 6249, 6253, 6275, 6276, 6283, 6426, 7488, 7494, 7497, 7512, 7714, 7725, 7730, 7733, 7735, 7748, 7749, 7768a, 7768b, 7773, 7775a, 7775b, 7777, 7782, 7786, 7814, 7828, 7829, 8014, 8015, 8044a, 8044b, 8123, 8135, 8154, 8595, 8659, 8728, 9555, 9557, 9567). To the best of our knowledge, all these 212 newly profiled miRNAs are reported for the first time in wheat. The 212 new miRNAs are resulted from the reference miRNAs of *O*. *sativa* (25%), *B*. *distachyon* (15%), *H*. *vulgare* (12%), *S*. *bicolor* (11%), *G*. *max* (6%), *M*. *truncatula* (6%), *P*. *trichocarpa* (3%), *P*. *persica* (2%), *A*. *thaliana* (2%), *V*. *vinifera* (2%), *B*. *rapa* (2%), *G*. *raimondii* (2%), *S*. *tuberosum* (2%), *C*. *sinensis* (1%), *G*. *hirsutum* (1%), *C*. *cardunculus* (1%), *C*. *papaya* (1%), *S*. *lycopersicum* (1%), *B*. *napus* (1%), *A*. *hypogaea* (0.5%), *A*. *auriculiformis* (0.5%), *A*. *trichopoda* (0.5%), *P*. *euphratica* (0.5%), *N*. *tabacum* (0.5%) and *H*. *brasiliensis* (0.5%). All the newly identified conserved wheat miRNAs considered as a valid candidates after fulfilling the empirical formula A, B and D for biogenesis and expression of the miRNAs, suggested by Ambros et al. [2]. Ambros and co-workers [2] also explained that only principle D is enough for homology sequences to validate as new miRNA in different plant species.

### 3.2. Wheat miRNAs characterization

**Fig 1:**
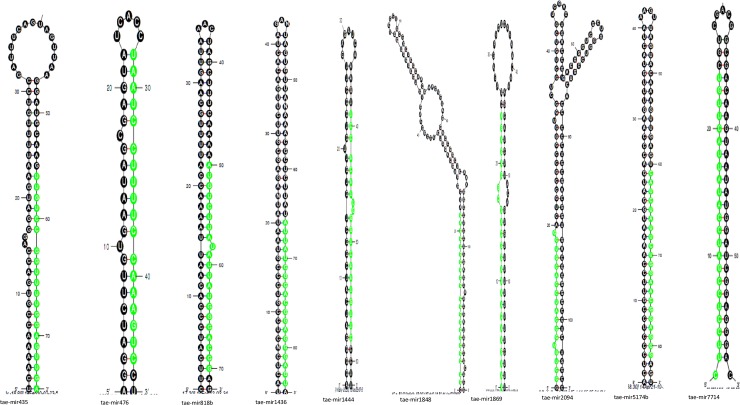
The newly identified wheat miRNAs’ secondary structures. The wheat pre-miRNAs’ secondary structures were developed through the Mfold algorithm. These structures clearly show the mature miRNAs in green, in the stem portion of the stem-loop structures.

The newly profiled wheat miRNAs were characterized and annotated in terms of pre-miRNAs length, MFE of pre-miRNAs, mature miRNA sequences with mismatches, number of mismatches, mature sequence length, ESTs, strand orientation, mature sequences arm, GC percentage and organ of expression (for detail, S2 Table). All the mature sequences of the new conserved wheat miRNAs are observed in the stem regions of the stem-loop structures, (some are shown in Fig 1). The predicted miRNAs’ stem-loop structures showed that there are at least 11–21 nucleotides engaged in Watson–Crick or G/U base pairings between the mature miRNA and the opposite arms (pre-miRNAs) in the stem region and the hairpin precursors do not contain large internal loops or bulges. Many researcher have been reported same results for the miRNAs in other plants and animals [6, 11, 12, 32].

The wheat pre-miRNAs based on length were observed in a range from 37 to 453 nt with an average of 107 nt. The classification, in order to pre-miRNAs lengths are found as, 1–50 nt (8 out of 212) pre-miRNA and made 4% of the total pre-miRNA, from 51–100 nt (118 out of 212) 56%, 101–150 nt (51 out of 212) 24%, 151–200 nt (24 out of 212) 11%, 201–300 nt (9 out of 212) 4%, 301–400 nt (1 out of 212) 0.4% and 401–500 nt (1 out of 212) 0.4%. In this research, MFE of the newly identified wheat pre-miRNAs were observed from -149 Kcal mol^-1^ to -9 Kcal mol^-1^ with an average of -36 Kcal mol^-1^. According to class boundaries -150 to -100 Kcal mol^-1^ (2) produced 1% of the total pre-miRNA, from ˗99 to -50 (44) 20% at the last -49 to -00 (166) 78% of all the pre-miRNAs. These results were supported by different researchers previously reported MFEs of pre-miRNAs in other organisms [9–11, 32]. The miRNAs hybridization with opposite arms is observed with minimum 43%, maximum 100% and an average of 74% engaged in Watson–Crick or G/U base pairings. According to class boundaries of matures hybridization with opposite arms, from higher to lower, are noted as 100% to 95% (5 out of 212) produced 2% of the total, 94% to 90% (13 out of 212) 6%, 89% to 80% (46 out of 212) 22%, 79% -70% (76 out of 212) 36%, 69% to 60% (55 out of 212) 26% and below 60% (17 out of 212) 8%. The miRNAs hybridization with opposite arms of the newly identified wheat miRNAs are found in agreement with the reported data of the researchers for other organisms [4, 9, 10, 21, 32].

Our study revealed that significant results regarding the total mismatches found in newly predicted wheat mature miRNAs with their reference sequences are from 1–4 with an average of two mismatches. Therefore, with three mismatches (69 miRNAs out of 212) are found 33% of the total miRNAs, two mismatches (57 miRNAs out of 212) with 27%, four mismatches (53 miRNA out of 212) with 25%, one mismatch (15 miRNA out of 212) with 7% and perfectly matched were 8% (18 miRNAs out of 212). These results of wheat miRNAs mismatches in a range of 0–4 are similar as reported for many plant and animal species [9–16, 32].

Wheat miRNAs’ mature lengths were found with minimum 18 nt and maximum 25 nt with an average of 22. According to class boundaries, mature sequences length ranges from lowest to highest are as, 18 nt have (2 out of 212) made 1% of total, 19 nt (7 out of 212) 3%, 20 nt (30 out of 212) 14%, 21 nt (88 out of 212) 42%, 22 nt (36 out of 212) 17%, 23 nt (17 out of 212) 8%, 24 nt (30 out of 212) 14% and 25 nt (2 out of 212) 1%. The wheat mature sequences length range is observed in agreement with the other known plant miRNAs [16, 32]. The current study revealed that strand orientation of the newly profiled 100 miRNAs out of 212 are found in sense strand that produced 47% of the total miRNAs. While 112 miRNAs out of 212 are found in anti-sense strand orientation that made 53% of the total miRNAs. The 113 out of 212 that made 53% of the total mature sequences are observed on the 5' arm of the stem-loop secondary structures, whereas, the 99 out of 212 (47%) are found on the 3' arm. GC percentage is an important parameter of characterization for a nucleotide sequence. The GC% of the newly predicted wheat miRNAs are found with 14% minimum, 90% maximum and an average of 47%. The detail GC % results shown in the form of class boundaries are, 10% to 40% (77 out of 212) 36%, 41% to 60% (98 out of 212) 46%, 61% to 80% (27 out of 212) 13%, 81% to 95% (10 out of 212) 4% of the total. The newly profiled wheat miRNAs based on their ESTs were also characterized for their organ of expression. The maximum miRNAs are found in root (56 out of 212) made 26% of the total and followed by leaf 17%, crown 13%, anther 8%, seedling 6.6%, shoot 6%, seed 5.6%, spike 5%, endosperm 2%, pistil 2%, kernel 1.4%, embryo 1.4%, florets 1%, ovary 1%, stem 1%, cultured 1%, callus 0.5%, and egg cell 0.5%. The organ based expression of wheat miRNAs will be helpful in devising better plant organ developing and regulation. The different organ based expression of the miRNAs reported through comparative genomics approaches are in agreements with the previous reports in other plant species [9–16, 32].

### 3.2 Amplification and validation of wheat miRNAs

**Fig 2:**
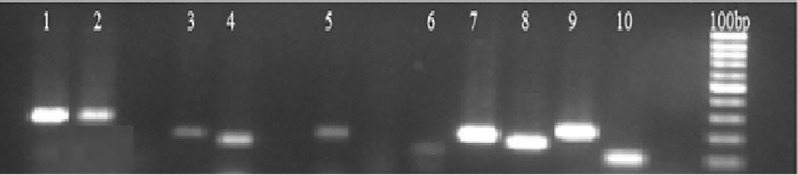
Wheat miRNAs RT-PCR expressional validation. One housekeeping gene and nine wheat miRNAs; 1 (Housekeeping gene Ta54227- AAA-superfam. ATPases), 2 (tae-miR5040), 3 (tae-miR6220), 4 (tae-miR169), 5 (tae-miR172d), 6 (tae-miR827), 7 (tae-miR5523), 8 (tae-miR530b), 9 (tae-miR530a) and 10 (tae-miR1522), were selected and subjected to RT-PCR expression analysis for the experimental validation. The product of each sample was separated on a 1.5% (w/v) agarose gel with 100 base pair DNA leader.

The RT-PCR analysis is important for experimental validation of the newly profiled wheat miRNAs. The nine wheat miRNAs along with the housekeeping gene Ta-54227 (AAA-superfam. ATPases) [24] were amplified by RT-PCR expressional analysis as shown in Fig 2 as; 1 (Ta-54227 housekeeping gene), 2 (tae-miR5040), 3 (tae-miR6220), 4 (tae-miR169), 5 (tae-miR172d), 6 (tae-miR827), 7 (tae-miR5523), 8 (tae-miR530b), 9 (tae-miR530a) and 10 (tae-miR1522). Six wheat miRNAs; tae-miR1522, tae- miR1439, tae-miR1858a, tae-miR2275c, tae-miR5502 and tae-miR7778 were not-validated through RT-PCR. It might be due to the difference in the developmental stage, environmental factor and/or wheat variety. Different researchers in various plant species [9–11] support these results.

### 3.3. Wheat miRNAs putative targets prediction

Targets prediction is an important step of annotation and characterization for the newly profiled wheat miRNAs. A total of 32927 target genes were identified for the newly predicted 212 new conserved wheat miRNAs (S3 Table) by a very stringent approach as described above. Based on gene ontology annotation, these targets comprises of 50 GO-terms (Table 1) and engaged in significant processes like; metabolism, transcription factors, transporter, cell signaling, structural protein, growth & development and stress related.

**Table 1:** Putative wheat targets enrichment analysis in GO-terms. Where, BP = Biological Process, MF = Molecular Function, CC = Cellular Component, and FDR = False Discovery Rates.

GO term	Ontology	Description	Gene Number	p-value	FDR
GO:0000003	BP	reproduction	107	5.00E-10	8.00E-07
GO:0008037	BP	cell recognition	99	9.50E-10	8.00E-07
GO:0009856	BP	pollination	99	9.50E-10	8.00E-07
GO:0022414	BP	reproductive process	107	5.00E-10	8.00E-07
GO:0048544	BP	recognition of pollen	99	9.50E-10	8.00E-07
GO:0009875	BP	pollen-pistil interaction	99	9.50E-10	8.00E-07
GO:0008535	BP	respiratory chain complex IV assembly	40	3.00E-09	2.10E-06
GO:0051704	BP	multi-organism process	100	1.70E-08	1.10E-05
GO:0032501	BP	multicellular organismal process	116	4.40E-07	0.00025
GO:0007154	BP	cell communication	147	1.90E-06	0.00094
GO:0015985	BP	energy coupled proton transport, down electrochemical gradient	70	0.00011	0.037
GO:0015986	BP	ATP synthesis coupled proton transport	70	0.00011	0.037
GO:0009142	BP	nucleoside triphosphate biosynthetic process	74	0.00012	0.037
GO:0009145	BP	purine nucleoside triphosphate biosynthetic process	74	0.00012	0.037
GO:0006754	BP	ATP biosynthetic process	70	0.00011	0.037
GO:0009206	BP	purine ribonucleoside triphosphate biosynthetic process	74	0.00012	0.037
GO:0009201	BP	ribonucleoside triphosphate biosynthetic process	74	0.00012	0.037
GO:0046034	BP	ATP metabolic process	76	0.00016	0.045
GO:0009152	BP	purine ribonucleotide biosynthetic process	83	0.00018	0.048
GO:0003682	MF	chromatin binding	272	1.30E-22	4.40E-19
GO:0043531	MF	ADP binding	483	7.20E-22	1.20E-18
GO:0003899	MF	DNA-directed RNA polymerase activity	146	3.70E-07	0.00042
GO:0030246	MF	carbohydrate binding	131	6.50E-07	0.00056
GO:0032549	MF	ribonucleoside binding	53	1.80E-06	0.001
GO:0005515	MF	protein binding	2072	1.80E-06	0.001
GO:0034062	MF	RNA polymerase activity	154	5.60E-06	0.0027
GO:0016820	MF	hydrolase activity, acting on acid anhydrides, catalyzing transmembrane movement of substances	174	2.30E-05	0.0098
GO:0005488	MF	binding	7160	2.70E-05	0.01
GO:0051540	MF	metal cluster binding	81	6.70E-05	0.021
GO:0051536	MF	iron-sulfur cluster binding	81	6.70E-05	0.021
GO:0045735	MF	nutrient reservoir activity	70	9.50E-05	0.027
GO:0009522	CC	photosystem I	45	4.80E-14	5.30E-11
GO:0015935	CC	small ribosomal subunit	28	8.80E-13	4.80E-10
GO:0005739	CC	mitochondrion	96	3.40E-11	1.30E-08
GO:0000276	CC	mitochondrial proton-transporting ATP synthase complex, coupling factor F(o)	33	5.90E-10	1.60E-07
GO:0045263	CC	proton-transporting ATP synthase complex, coupling factor F(o)	62	1.30E-09	2.80E-07
GO:0005753	CC	mitochondrial proton-transporting ATP synthase complex	33	9.30E-09	1.70E-06
GO:0033177	CC	proton-transporting two-sector ATPase complex, proton-transporting domain	75	1.60E-07	2.60E-05
GO:0044429	CC	mitochondrial part	71	7.30E-07	9.20E-05
GO:0031224	CC	intrinsic to membrane	819	7.60E-07	9.20E-05
GO:0005740	CC	mitochondrial envelope	64	8.90E-07	9.80E-05
GO:0031966	CC	mitochondrial membrane	60	1.60E-06	0.00015
GO:0016020	CC	membrane	1943	1.60E-06	0.00015
GO:0044455	CC	mitochondrial membrane part	46	1.90E-06	0.00016
GO:0016021	CC	integral to membrane	800	2.50E-06	0.00019
GO:0005743	CC	mitochondrial inner membrane	50	2.60E-06	0.00019
GO:0033279	CC	ribosomal subunit	32	3.30E-06	0.00023
GO:0019866	CC	organelle inner membrane	51	5.00E-06	0.00033
GO:0045259	CC	proton-transporting ATP synthase complex	67	6.30E-05	0.0039
GO:0044425	CC	membrane part	1022	0.00027	0.015

**Fig 3:**
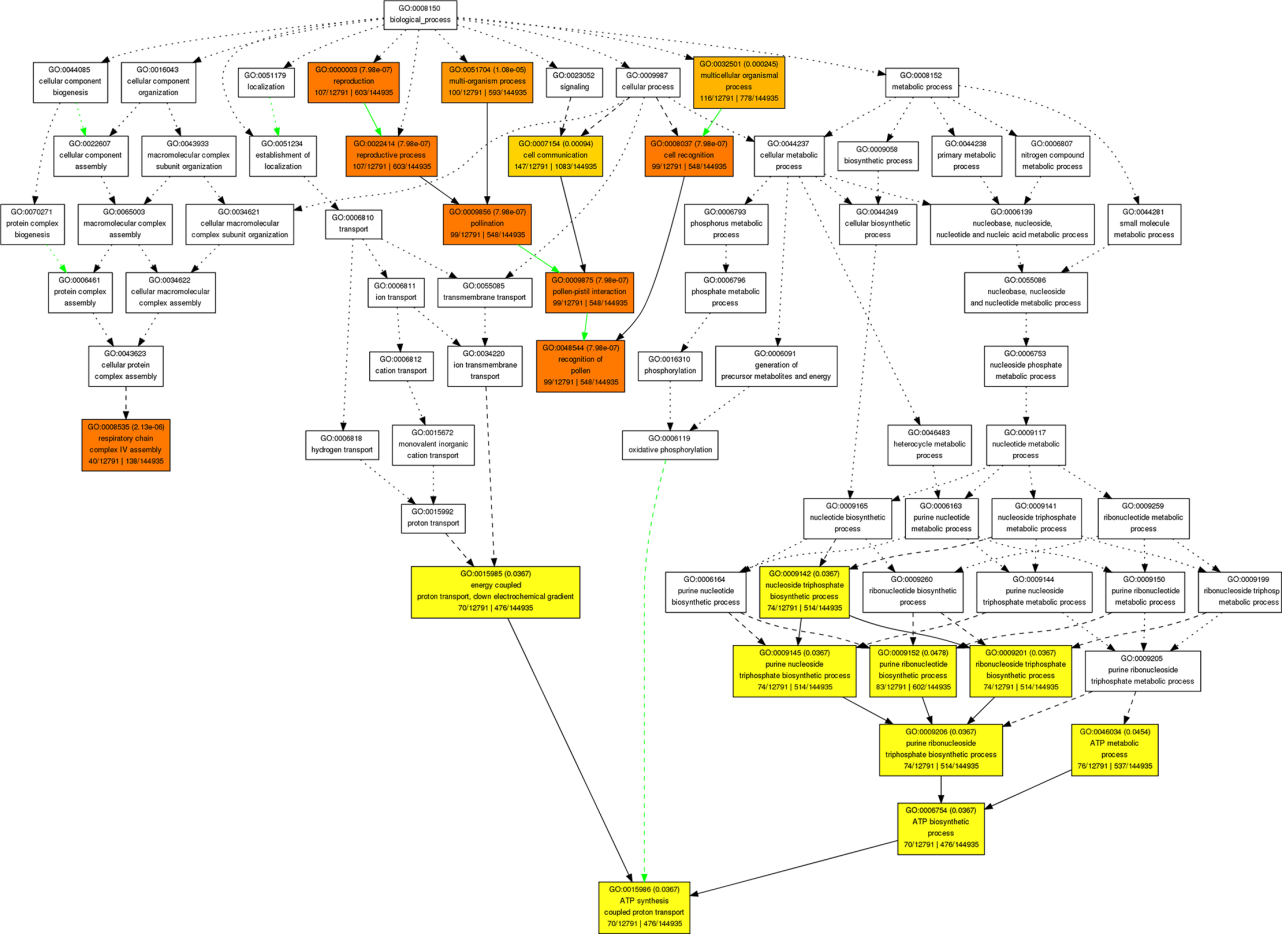
GO-biological processes. Based on agriGo, more complicated enriched biological processes was built and wheat miRNAs are involved in many different biological processes, mainly in reproduction, multi-organism process, cell communication, cell recognition, respiration and biosynthesis processes.

GO-biological process revealed that the putative targets of the newly profiled wheat miRNAs are significantly involved in reproduction (GO:0000003), multicellular (GO:0032501) and multi-organisms (GO:0051704) processes, cell communication (GO:0007154), cell recognition (GO:0008037), respiratory chain complex IV assembly (GO:0008535) and nucleoside triphosphate biosynthetic process (GO:0009142) as illustrated in Fig 3. Respiratory chain complex IV is also known as cytochrome c oxidase (COX). It is the terminal member of the respiratory chain of the mitochondrion and is responsible for transferring electrons to oxygen, the final acceptor, in the classical respiratory pathway [33]. Dahan et al. [34] studied Cytochrome C Oxidase deficient1 (COD1) gene containing *Arabidopsis thaliana* plants and found that this gene is involved in a late embryo development arrest leading to seed abortion. They also demonstrated that by growing homozygous cod1 plantlets in vitro have shown a complete loss of mitochondrial respiratory complex IV. Garcia et al. [35] studied *Arabidopsis thaliana* AtCOX17 genes, that contributes in the transfer of copper for COX assembly and found that AtCOX17 genes are induced by several stress conditions and abscisic acid. This means the newly identified miRNAs targeting wheat COX genes would help us to understand and manage it against several stress conditions.

In the reproduction process (GO:0000003), recognition of pollen (GO:0048544) and oogenesis (GO:0048477) are identified and annotated as a potential targets of the tae-miR1535, tae-miR3476, tae-miR5386, tae-miR5783, tae-miR8154, tae-miR6276, tae-miR6249, tae-miR6111, tae-miR6202, tae-miR3627 and tae-miR8044b. Male and female gametes are the specialized structures developed by flowering plants. They have crucial role in seeds and fruit production. Understanding of male fertility through pollen recognition and production genes can be utilized for the development of novel hybrid seed production systems in wheat [36]. The wheat’s miRNAs, identified in this study, targeting genes involved in the processes of male, female gametes and reproduction would be a good source to enhance seed productions and regulate male fertility in wheat.

In multicellular organismal process (GO:0032501), protein amino acid phosphorylation (GO:0006468), ubiquitin-dependent protein catabolic process (GO:0006511), multicellular organismal development (GO:0007275), G2/M transition of mitotic cell cycle (GO:0000086) and regulation of meristem structural organization (GO:0009934) are predicted as a putative targets of the wheat miRNAs; tae-miR1438, tae-miR6184, tae-miR7748, tae-miR7749, tae-miR8154 and tae-miR9557. Ubiquitination is an important biological event in plants that is involved in the regulation of various biological processes such as growth and development, response to biotic and abiotic stresses and regulation of chromatin structure [37]. The results in this study showing wheat miRNAs targeting ubiquitin related genes have potentials to fine-tune the wheat plant for better production through managing growth and development as well as biotic and abiotic stresses.

In cell communicational process (GO:0007154), the significant potential targets of wheat miRNAs are; protein kinase activity (GO:0004672), regulation of Rab GTPase activity (GO:0032313), Rab GTPase activator activity (GO:0005097), regulation of ARF protein signal transduction (GO:0032012), ARF guanyl-nucleotide exchange factor activity (GO:0005086) and regulation of signal transduction (GO:0009966). These identified as potential targets of tae-miR477a, tae-miR2088a, tae-miR2905, tae-miR4995, tae-miR5075, tae-miR5076, tae-miR5169a, tae-miR5225, tae-miR6111, tae-miR6189, tae-miR6207, tae-miR6214, tae-miR6233 and tae-miR6276. Rab proteins belong to the small guanosine triphosphatases (GTPases) superfamily. Rabs act as molecular switches, which play a vital role in both endocytic and exocytic traffic in eukaryotic cells, being active in their GTP-bound state and inactive in their GDP-bound state [38]. Liu et al. [39] reported that wheat TaRab7 plays an important role in the early stage of wheat-stripe rust fungus interaction and in stress tolerance. The identification of such gene as a potential target of newly profiled wheat miRNAs would enabled researchers to fight, cope and manage the rust fungal attack by wheat crops.

**Fig 4:**
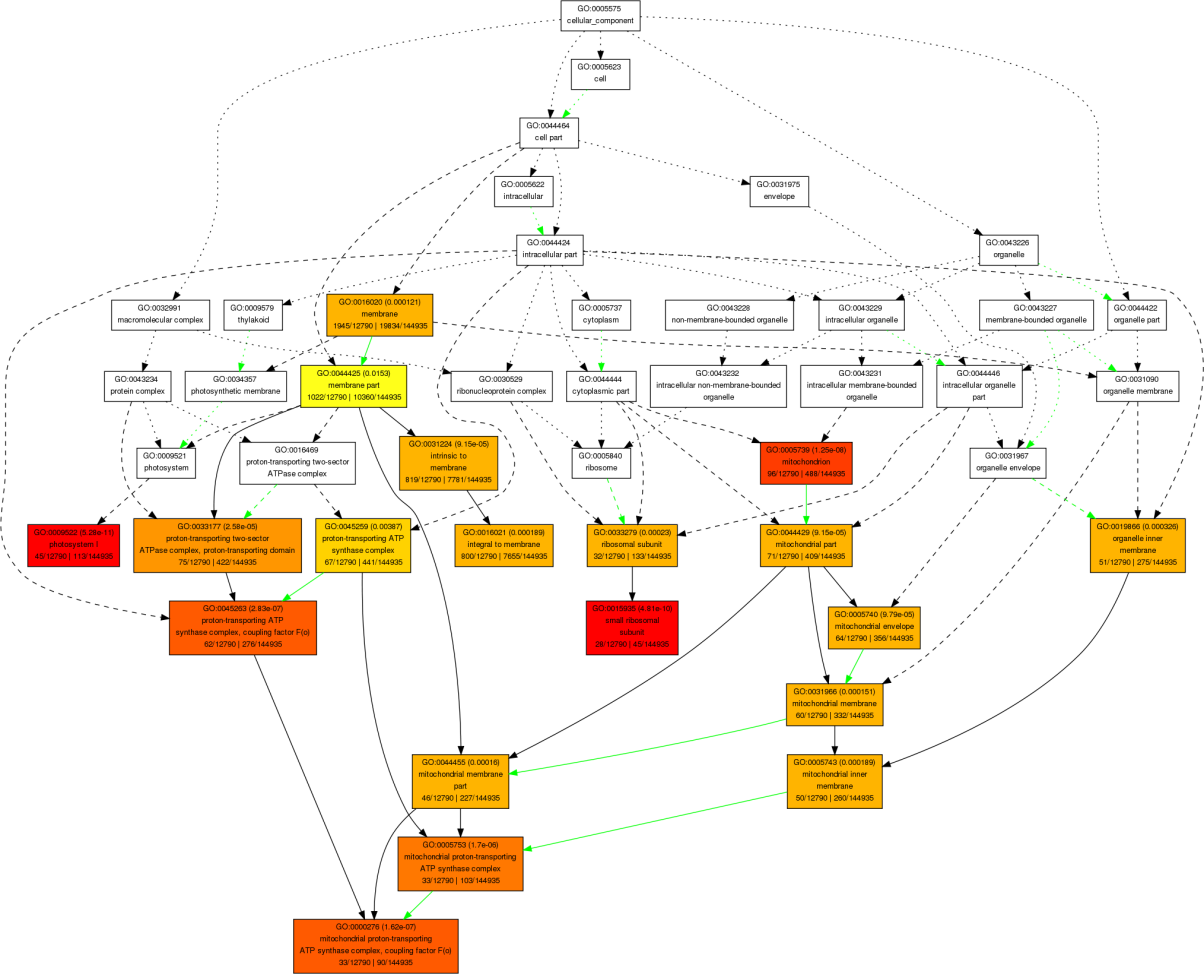
GO-cellular processes. Based on agriGo, more complicated enriched cellular component processes was built and wheat miRNAs are involved in many different cellular components, mainly in membrane and membrane-linked, mitochondrion and small ribosomal subunit.

**Fig 5:**
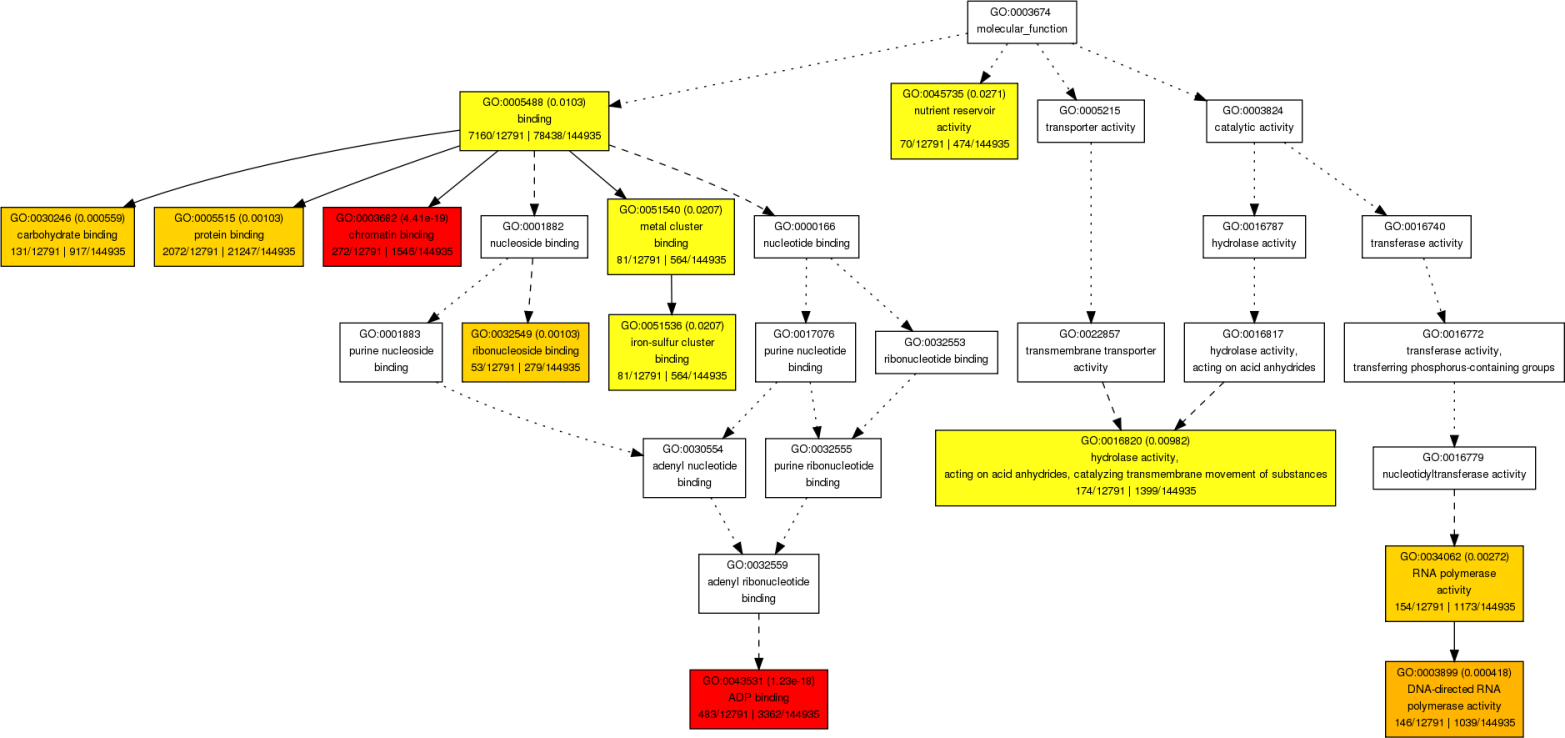
GO-molecular functions. Based on agriGo, more complicated enriched molecular functional processes was built and wheat miRNAs are involved in many different molecular functions, mainly in bindings, nutrient reservoir activity, hydrolase activity and in RNA polymerase activity.

Based on the GO-cellular component analysis the significant numbers of targeted genes are involved in the membrane (GO:0016020), mitochondrion (GO:0005739), ribosomal subunit (GO:0033279), and organelle inner membrane (GO:0019866) (for detail Fig 4). Similarly, GO- molecular functional annotation revealed that significant numbers of the targeted genes are engaged in the process of binding (GO:0005488) such as; carbohydrate binding (GO:0030246), protein binding (GO:0005515), chromatin binding (GO:0003682) ribonucleoside binding (GO:0032549) and metal cluster binding (GO:0051540) along with hydrolase activity (GO:0016820) and DNA directed RNA polymerase activity (GO:0003899) as illustrated in Fig 5. In the process of binding (GO:0005488), some potential wheat miRNAs targets are; protein binding (GO:0005515), metal ion binding (GO:0046872), DNA replication (GO:0006260), ATP binding (GO:0005524), DNA binding (GO:0003677), regulation of transcription, (GO:0006355), transcription factor activity (GO:0003700), zinc ion binding (GO:0008270) nucleic acid binding (GO:0003676), lipid binding (GO:0008289), sequence-specific DNA binding (GO:0043565), single-stranded telomeric DNA binding (GO:0043047), telomere maintenance (GO:0000723), zinc ion binding (GO:0008270), polysaccharide binding (GO:0030247), calcium ion binding (GO:0005509), transcription factor activity (GO:0003700), GTP binding (GO:0005525) and copper ion binding (GO:0005507). These potential bindings related genes are targeted by wheat miRNAs such as; tae-miR1861b, tae-miR2118a, tae-miR3636, tae-miR5034, tae-miR5056, tae-miR5076, tae-miR5083, tae-miR5203, tae-miR5234, tae-miR5543, tae-miR5568c, tae-miR5641, tae-miR5721, tae-miR6164, tae-miR6207, tae-miR7777, tae-miR7829 and tae-miR8014. The GO-molecular function based enrichment analysis also revealed that some potential putative wheat miRNAs target genes have a role in cell transport activity. Such targets are; transmembrane transporter activity (GO:0022857), transmembrane transport (GO:0055085), transporter activity (GO:0005215), cation transport (GO:0006812), cation transmembrane transporter activity (GO:0008324), mitochondrial proton-transporting ATP synthase complex, coupling factor F(o) (GO:0000276), hydrogen ion transmembrane transporter activity (GO:0015078), metal ion transmembrane transporter activity (GO:0046873), inorganic anion exchanger activity (GO:0005452), anion transport (GO:0006820), potassium ion transmembrane transporter activity (GO:0015079), glycolipid transport (GO:0046836), magnesium ion transmembrane transporter activity (GO:0015095) and chloride transport (GO:0006821).

A number of newly profiled wheat miRNAs are predicted as a regulator of these cell transport related targets, such as; tae-miR818b, tae-miR2643, tae-miR3522, tae-miR5265, tae-miR5298, tae-miR5490, tae-miR5783, tae-miR6116, tae-miR6181, tae-miR6188, tae-miR6276, tae-miR6426, tae-miR7488 and tae-miR7749. These miRNAs targeting genes involved in transport-related activities would be a good source to fine tune the wheat for desirable traits by reprogramming the wheat cell transport processes.

Magnesium (Mg) is the second most abundant cation in plants. It plays a significant role in many physiological and biochemical processes like photosynthesis, enzyme activation, and synthesis of nucleic acids and proteins [40]. Mg also serves as a regulator of cation-anion balance in cells and as an osmotically active ion regulating cell turgor together with potassium (K) [41]. To maintain an optimal Mg level in various tissues, plants have evolved efficient transport and regulation machinery for Mg^2+^ distribution throughout the whole plant [42]. The newly predicted wheat miRNAs including tae-miR5167a, tae-miR5543, tae-miR5568c, tae-miR6035, tae-miR6224a, tae-miR7768b and tae-miR8659 are found to target the Mg transport associated genes such as, magnesium ion transport (GO:0015693), magnesium ion transmembrane transporter activity (GO:0015095) and magnesium ion binding (GO:0000287). These tae-miRNAs would be useful to regulate Mg accumulation in the plant for a better management of photosynthesis, enzymes activation, nucleic acid and protein synthesis.

The salinity of soil is one of the main abiotic stresses that limit agricultural yields global and at least 50% of total agricultural lands are at risk of salinization. The most comprehensively studied gene class in relation to salinity stress physiology is the family of cation/proton antiporter 1 [43]. Here, the wheat miRNAs as; tae-miR435, tae-miR827, tae-miR1522, tae-miR5167a, tae-miR5490, tae-miR6180 tae-miR6191b, tae-miR6275, tae-miR7714 and tae-miR7768b are engaged to target the two antiporter gene like; antiporter activity (GO:0015297) and solute:hydrogen antiporter activity (GO:0015299). These tae-miRNAs could be used to reprogrammed cation/proton antiporter activities and enhance the salinity tolerance in wheat.

Plant growth, development, biotic and abiotic stress responses are also regulated by MAP kinase phosphatases (MKPs). They are the major regulators of MAPK signaling pathways and play vital roles in plant survival and sustainability. Various phosphorylation and kinase-associated genes are the prominent players of MAPK signaling pathways [44]. A number of such genes as, protein amino acid phosphorylation (GO:0006468), protein kinase activity (GO:0004672), ATP binding (GO:0005524), regulation of Rab GTPase activity (GO:0032313) Rab GTPase activator activity (GO:0005097), regulation of signal transduction (GO:0009966), phosphoprotein phosphatase inhibitor activity (GO:0004864), regulation of phosphoprotein phosphatase activity (GO:0043666) identified as potential targets of newly profiled wheat miRNAs, including tae-miR477b, tae-miR827, tae-miR1435a, tae-miR1438, tae-miR1522, tae-miR1861b, tae-miR3476, tae-miR5641, tae-miR8135 and tae-miR8154. Thus, wheat growth, development, biotic and abiotic stress resistance can be devised by managing these cell signaling players through the newly predicted wheat miRNAs.

The well-known targeted proteins class of miRNAs is transcription factor, reported in nearly all plants and animals [9–16, 32]. The newly identified wheat conserved miRNAs, as tae-miR1435b, tae-miR1436, tae-miR1858, tae-miR5508, tae-miR5565h, tae-miR5814, tae-miR6026, tae-miR6189, tae-miR6202 and tae-miR6233 are also found to target such significant genes like, nucleotide binding (GO:0000166), DNA binding (GO:0003677), DNA replication (GO:0006260), cofactor binding (GO:0048037), RNA-directed DNA polymerase activity (GO:0003964), RNA-dependent DNA replication (GO:0006278), RNA binding (GO:0003723), nucleic acid binding (GO:0003676), ATP-dependent helicase activity (GO:0008026), helicase activity (GO:0004386), transcription, DNA-dependent (GO:0006351) and transcription factor activity (GO:0003700). These findings would be helpful to fine-tune the wheat for better traits and yields.

The transcription factor MYB performs a vital role in abiotic stress responses. In Arabidopsis, AtMYB96 overexpressed plants have shown dehydration tolerance by participating the ABA and auxin signaling pathways, as well as participated in improving freezing and drought tolerance by regulating a lipid transfer protein 3 [45, 46]. Similarly, AtMYB44 and AtMYB60 integerate in plant responses to dehydration stress by controlling stomatal openings [47, 48]. Wei et al. [49] cloned the wheat TaODORANT1, a R2R3-type MYB transcription factor gene in tobacco and found that TaODORANT1 was up-regulated under high salinity, PEG6000, H_2_O_2_, and ABA treatments. This TaODORANT1 overexpression improved drought and salt tolerance in transgenic tobacco plants. The newly identified wheat miRNA tae-miR858 is found to target the MYB-transcription factor. This would serve a potential resource to manage the wheat under biotic and abiotic stresses and to regulate it for better crop production.

Another important transcription factor is WRKY that play vital roles in plant resistance responses to pathogens. Wang et al. [50] identified that the two WRKY genes TaWRKY49 and TaWRKY62 were originally in association with high-temperature seedling-plant resistance to wheat stripe rust, caused by the fungal pathogen Puccinia striiformis f. sp. tritici (Pst) resistance in wheat. The wheat miRNA, tae-miR5082 identified in this study is found to target the transcription factor WRKY. With the help of such wheat miRNA, fungal rust resistance could be better managed and that would ultimately increase the crop production.

Another significant transcription factor zinc finger are reported to performs cricial roles in several plant processes including regulation of growth and development, signaling networks, responses to environmental stresses. Recently, Agarwal and Khurana [51] identified and explored the involvement of wheat zinc finger (TaZnF) in plant stress response, mainly heat stress. They reported that the overexpression of TaZnF in Arabidopsis transgenics have showed considerable tolerance to cold and oxidative stress. Based on these observations, they suggested that TaZnF acts as a positive regulator of thermal stress and thus can be of great significance in understanding and improving temperature stress tolerance in plants. As the transcription factor, zinc finger is predicted as putative potential target gene of two newly profiled wheat miRNAs (tae-miR5183 and tae-miR5562), this would be useful to better program the wheat resistance under heat, cold and oxidative stress.

## Conclusions

The 212 new conserved miRNAs belonging to 185 families from wheat EST sequences were identified by applying comparative genomics approaches. All these miRNAs are reported for the first time in wheat. In addition, for these 212 wheat miRNAs, 32927 targets are predicted which have roles in 50 GO-enrichment pathways. The targets are found to involve in different processes, as metabolism, transcription factor, transporter, cell signaling, structural protein, stress-related and growth & development. Some randomly selected wheat miRNAs are also validated by RT-PCR. In detail, characterization and annotation of the newly profiled miRNAs and their targets were also done. These results will contribute to wheat stress-resistant breeding as well as understanding better yield’s traits.

## Supporting information

S1 Table The wheat pre-miRNAs primer sequences for RT-PCR experimental validation. Fifteen randomly selected wheat miRNAs subjected to expression analysis through RT-PCR are given here with melting temperature (Tm) primers, product size (bp) and source EST.
(DOCX)

S2 Table Annotation of newly profiled wheat conserved miRNAs. The wheat predicted miRNAs are characterized in terms of source miRNAs, precursor miRNA length (PL), minimum free energy (MFE), mature sequences (MS), number of mismatches (represented in bold and red) NM, mature sequence length (ML), source EST (SE), mature sequence arm (MSA), GC percentage (GC%), strand orientation (SO) and organ of expression (OE).
(DOCX)

S3 Table Wheat miRNAs targets. The wheat’s miRNA families and their putative targets are predicted with the help of psRNATarget. The targeted proteins name, Genbank Acc., Hybridization results and functions are provided here.
(TXT)
