# Supplementary material for: Identification and annotation of newly conserved microRNAs and their targets in wheat (Triticum aestivum L.)
Source: PLoS One. 2018 Jul 10;13(7):e0200033. doi: 10.1371/journal.pone.0200033 (PMC6038988; doi:10.1371/journal.pone.0200033)
Supplement: S1 Table — Fifteen randomly selected wheat miRNAs subjected to expression analysis through RT-PCR are given here with melting temperature (Tm) primers, product size (bp) and source EST. (DOCX) [file pone.0200033.s001.docx]

**S1 Table. The wheat pre-miRNAs primer sequences for RT-PCR experimental validation.** Fifteen randomly selected wheat miRNAs subjected to expression analysis through RT-PCR are given here with melting temperature (Tm) primers, product size (bp) and source EST.

| Wheat miRNAs | Tm | Primers | Product size | EST |
| --- | --- | --- | --- | --- |
| tae-MIR-1522 | 60.41  60.62 | TGCAGGTCATGCTACCAGAA  ATGCCTCCTGGATCTGTCAA | 220 | HX107438.1 |
| tae-Mir-5040 | 60.52  60.56 | CGCAATGGATTGTCAACGTA  ATCTGCCTGAGCTGTTCGAC | 237 | HX080066.1 |
| tae-Mir-6220 | 59.83  60.20 | TGGCATCTGCTGTCGAATAC  ACACCACTTGCACAACTCCA | 154 | HX083836.1 |
| tae-Mir169a | 59.70  59.84 | GGTAGCCAAGGATGACTTGC  GCTGGCATGAAGGGTAAGAG | 130 | HX077861.1 |
| Tae-Mir-172d | 59.78  60.25 | CGCGTAGCCACCTACATACA  AACATTGGCATGTGTCATGG | 182 | HX083757.1 |
| Tae-Mir-1439 | 57.19  59.69 | AATAATATGGTACTCCATCCAAAAA  TCAATGACTTGGGTTTGCTG | 150 | HX094398.1 |
| Tae-Mir-1858a | 57.91  60.49 | AGATGTAGCACCCGAAAGC  CTCCTCTCTCCCCGTAGCTC | 153 | HX103445.1 |
| Tae-Mir-2275c | 60.28  59.44 | ATGCAGCTACTTCCCAGGTG  CAACCGAAGGTGAGATATTGG | 161 | HX083889.1 |
| Tae-Mir-5502 | 57.89  58.60 | TCTCTTGTAAAATACATTAGTGGGAAT  ATATAAACGTATCCCCGTATCCA | 150 | HX101882.1 |
| Tae-Mir-5523 | 59.90  59.99 | TAGCAAAACCGGTAGCGACT  TTTTTGTTTCGGTATCTCTGGAA | 184 | HX094480.1 |
| Tae-Mir-827 plus strand | 57.61  61.07 | TGGTTAGATGACCATCAGCA  CTACCTTCACCCGCGTCAT | 125 | HX116829.1 |
| Tae-Mir-7778 cluster | 61.49  60.03 | ATGGCAGGGAGCATCATGT  GGACACAGACAGCTCGTCAA | 177 | HX115168.1 |
| Tae-MIR530 plus strand | 59.20  59.97 | GTGCACAGCAAAGGGTTATG  GGGGAGAATGTGTGACGAGT | 210 | CA655063.1 |
| Tae-MIR530 minus strand | 59.97  61.26 | GGGGAGAATGTGTGACGAGT  GAAGAAAGGTCGAGGGCTTG | 154 | CA655063.1 |
| Tae-MIR1522 | 60.05  60.47 | TTTCACGCTGTTATCACCTTTTT  AGCCAGGTGGCATAAAATGA | 171 | HX107438.1 |
