# Supplementary material for: Identification and annotation of newly conserved microRNAs and their targets in wheat (Triticum aestivum L.)
Source: PLoS One. 2018 Jul 10;13(7):e0200033. doi: 10.1371/journal.pone.0200033 (PMC6038988; doi:10.1371/journal.pone.0200033)
Supplement: S2 Table — The wheat predicted miRNAs are characterized in terms of source miRNAs, precursor miRNA length (PL), minimum free energy (MFE), mature sequences (MS), number of mismatches (represented in bold and red) NM, mature sequence length (ML), source EST (SE), mature sequence arm (MSA), GC percentage (GC%), strand orientation (SO) and organ of expression (OE). (DOCX) [file pone.0200033.s002.docx]

| ***T. aestivum***  **S2 Table. Annotation of newly profiled wheat conserved miRNAs.** The wheat predicted miRNAs are characterized in terms of source miRNAs, precursor miRNA length (PL), minimum free energy (MFE), mature sequences (MS), number of mismatches (represented in bold and red) NM, mature sequence length (ML), source EST (SE), mature sequence arm (MSA), GC percentage (GC%), strand orientation (SO) and organ of expression (OE).  **miRNAs** | **Source**  **miRNAs** | **PL** | **MFE** | **MS** | **NM** | **ML** | **SE** | **MSA** | **GC%** | **SO** | **OE** |
| --- | --- | --- | --- | --- | --- | --- | --- | --- | --- | --- | --- |
| tae-miR413 | ath-miR413 | 63 | -14.70 | **GG**AGUUUCUCUUGUUCUGCA**G** | 3 | 21 | CK201601 | 3' | 48 | - | Root |
| tae-miR435 | osa-miR435 | 75 | -19.30 | UU**G**UCCGGU**U**UUGGAGUUGA | 2 | 20 | CA730042 | 3' | 45 | - | Pistil |
| tae-miR476 | hbr-miIR476 | 47 | -10.20 | UAAUCCUU--UU**C**CAAAGUC | 3 | 18 | HX073711 | 3' | 33 | + | Leaf |
| tae-miR477a | sly-miR477 | 101 | -35.98 | **CC**UCUCUCCCUCA**CC**GGCUCC | 4 | 21 | HX158207 | 5' | 71 | + | Root |
| tae-miR477b | ptc-miR477a | 205 | -47.79 | AUCUCCCUCAGAGGCUU**GUGU** | 4 | 21 | CA486622 | 3' | 52 | + | Anther |
| tae-miR529 | osa-miR529b | 54 | -15.70 | AGAAGAGAGAGA**UC**AC**U**GCU**G** | 4 | 21 | CD896805 | 5' | 48 | - | Seed |
| tae-miR815 | osa-miR815b | 140 | -34.60 | AAGGG**C**AUU**A**AGGAGAUUGGG | 2 | 21 | CX536018 | 5' | 48 | + | Spike |
| tae-miR818a | osa-miR818a | 81 | -16.40 | AAU**G**C**U**CUUAUAUUAUGGGACGG | 2 | 23 | HX039463 | 3' | 39 | - | Root |
| tae-miR818b | osa-miR818b | 72 | -17.20 | AAU**GU**CUUAUAUUAUGGGACGG | 2 | 22 | BJ272820 | 3' | 36 | - | Pistil |
| tae-miR827 | bdi-miR827 | 74 | -15.70 | UUAGAUGACCAUCAGCAAACA | 0 | 21 | HX116829 | 5' | 38 | + | Leaf |
| tae-miR854 | ath-miR854a | 132 | -52.40 | GAUGAGGA**C**AGGGAGGAGGA**C** | 2 | 21 | HX130162 | 3' | 62 | - | Root |
| tae-miR858 | ppe-miR858 | 143 | -46.50 | CUCGUUGUCUGUUCG**U**CCU**GG** | 3 | 21 | GH723028 | 3' | 57 | - | Leaf |
| tae-miR1428 | osa-miR1428a | 78 | -14.70 | UAAGAU**C**AAG**U**CGUG**U**AUUUG | 3 | 21 | CA610338 | 3' | 33 | + | Root |
| tae-miR1432 | sbi-miR1432 | 125 | -43.10 | **A**UCAGGAGAGAUGACACCGAC | 1 | 21 | CK195521 | 5' | 52 | - | Root |
| tae-miR1435a | sbi-miR1435a | 215 | -61.24 | UUUCU**A**AAGU**G**AAACUUU**A**C | 3 | 20 | DR739617 | 5' | 25 | + | Crown |
| tae-miR1435b | sbi-miR1435b | 138 | -51.40 | U**G**UCUUAAGUCAAAC**U**U**AG**U | 4 | 20 | DR735754 | 5' | 30 | + | Crown |
| tae-miR1435c | sbi-miR1435b | 138 | -41.45 | U**G**UCUUAAGUCAAACCUU**CA** | 3 | 20 | DR735754 | 5' | 35 | - | Crown |
| tae-miR1436 | osa-miR1436 | 85 | -58.30 | **AU**AUUAUGGGACGGAGGGAGU | 2 | 21 | CJ939471 | 3' | 48 | + | Seedling |
| tae-miR1437 | osa-miR1437b | 156 | -55.14 | G**A**GC**C**GGCGAGCUCCG**C**UGCCGC**C** | 4 | 24 | HX138375 | 3' | 83 | - | Root |
| tae-miR1438 | osa-miR1438 | 82 | -21.10 | AGGGUAAUU**A**UA-**A**AUUUUUAA | 3 | 21 | CK218082 | 5' | 14 | + | Crown |
| tae-miR1439 | osa-miR1439 | 88 | -35.00 | UUUUGG**G**ACGGAG**G**GAGUA**C**UA | 3 | 22 | HX094398 | 3' | 50 | + | Leaf |
| tae-miR1444 | ptc-miR1444a | 62 | -21.00 | UCCACAUU**G**GGUCAAUGUUC | 1 | 20 | CA632737 | 3' | 45 | + | Leaf |
| tae-miR1445 | ptc-miR1445a | 111 | -27.33 | -CC**U**UUGUAGCCUAGAAAAA | 3 | 19 | HX185932 | 5' | 37 | + | Root |
| tae-miR1516 | gma-miR1516b | 244 | -61.20 | AGCUUCUCU**C**CAGA**GC**ACUAU | 3 | 21 | CJ824131 | 3' | 48 | - | Root |
| tae-miR1522 | gma-miR1522 | 94 | -16.10 | UUUCUUGCUUAAAAUGAA**G**U | 2 | 20 | HX107438 | 3' | 25 | - | Leaf |
| tae-miR1535 | gma-miR1535b | 51 | -15.90 | **G**UUGUUUGUGGUGAUGU**G**UAG | 2 | 21 | CB412177 | 5' | 43 | - | Leaf |
| tae-miR1848 | osa-miR1848 | 119 | -58.00 | C**G**UCGCCGGCGCGCGCGUG**G**A | 2 | 21 | AL810411 | 5' | 86 | + | Endosperm |
| tae-miR1858 | osa-miR1858a | 56 | -19.43 | GAG**G**GGAGGACGGAG**C**GGGGC | 2 | 21 | HX103445 | 3' | 81 | - | Leaf |
| tae-miR1861a | osa-miR1861c | 170 | -59.28 | CGAUCUUG**GG**GCAAGAACUGAG | 2 | 22 | BG263159 | 5' | 55 | - | Spike |
| tae-miR1861b | osa-miR1861d | 55 | -23.10 | UGGUCUUGAGGCAGG**U**AC**A**UGAG | 2 | 23 | HX024507 | 5' | 52 | - | Shoot |
| tae-miR1866 | osa-miR1866 | 84 | -18.70 | UGAAAU**GU**CUGUAAAAUUCUUG | 2 | 22 | CJ888819 | 5' | 27 | - | Seedling |
| tae-miR1869 | osa-miR1869 | 69 | -10.20 | **AA**AGAACAAUAGGCAUG**A**GAGG**C**A | 4 | 24 | CJ796550 | 5' | 42 | + | Root |
| tae-miR1878 | bdi-miR1878 | 95 | -19.50 | AUUU**U**UAGUGUUCAGAU**A**GAGUU**A** | 3 | 24 | CK209741 | 5' | 25 | + | Crown |
| tae-miR1882 | osa-miR1882c | 47 | -10.80 | **G**GAUUGCUUUCAAGG**C**CAUUU**AA**U | 4 | 24 | CK168141 | 5' | 38 | - | Crown |
| tae-miR1913 | peu-miR1913 | 122 | - 45.60 | **CC**GGUCGGGGAUUGCAA**U**GGAG | 3 | 22 | CD875432 | 5' | 64 | + | Leaf |
| tae-miR2086 | aau-miR2086 | 75 | -27.60 | GACAUGAAUGCAG**GC**CUGGAA | 2 | 21 | DR736497 | 5' | 52 | + | Crown |
| tae-miR2088a | mtr-miR2088 | 169 | -54.10 | UCCAAUGUAA**C**C**A**AGG**C**CUA | 3 | 20 | CA502647 | 3' | 45 | - | Anther |
| tae-miR2088b | mtr-miR2088 | 199 | -63.35 | AGGCCU**U**G**G**UUACAUUGGAC | 2 | 20 | CA502647 | 5' | 50 | + | Anther |
| tae-miR2094 | osa-miR2094 | 107 | -49.00 | **GACG**GCUGUGGCAUCCACGUCG | 4 | 22 | HX188977 | 5' | 68 | - | Root |
| tae-miR2104 | osa-miR2104 | 65 | -34.30 | GCGGCGAG**C**GGA**G**GCGAGCG**CU** | 4 | 22 | BE400606 | 3' | 82 | + | Florets |
| tae-miR2106 | osa-miR2106 | 51 | -12.90 | CCGAGGUUUU**G**UGG**G**UACAUU | 2 | 21 | CK170538 | 5' | 48 | - | Crown |
| tae-miR2118a | osa-miR2118b | 98 | -33.70 | U**-**CCC**A**AUGCCUCCCAU**GA**CUA | 4 | 21 | CJ516205 | 3' | 52 | - | Spike |
| tae-miR2118b | osa-miR2118f | 96 | -32.20 | UUCC**CA**AUGCCUCCCAU**GA**CUA | 4 | 22 | CJ507504 | 3' | 50 | - | Spike |
| tae-miR2118c | osa-miR2118h | 130 | -37.50 | UUCCUGAUGCCUCUCAU**G**CCUA | 1 | 22 | CJ796550 | 3' | 50 | - | Root |
| tae-miR2118d | osa-miR2118o | 130 | -39.30 | -UCCUGAUGCCUC**U**CA**U**GCCUA | 3 | 21 | CJ796550 | 3' | 52 | - | Root |
| tae-miR2122 | osa-miR2122 | 55 | -13.10 | **C**UUCAAAAAUAACCU**G**UU**U**UUC | 3 | 22 | CJ804236 | 3' | 27 | - | Root |
| tae-miR2636 | mtr-miR2636 | 165 | -51.35 | **G**UUGG**G**U**U**GUGUGCUGAAUAU | 3 | 21 | BJ300674 | 5' | 43 | - | Spike |
| tae-miR2643 | mtr-miR2643a | 230 | -52.29 | UUUG**UA**AUCAGAAAUUAGAGA | 2 | 21 | CD867502 | 5' | 24 | - | Root |
| tae-miR2905 | osa-miR2905 | 52 | -12.50 | **C**ACAUGUCAGUGA**CC**CAAAGGCA | 3 | 23 | AL815763 | 5' | 52 | + | Embryo |
| tae-miR2924 | osa-miR2924 | 180 | -92.40 | **A**UCGC**CG**GCUCCGGCCGC**G**AC | 4 | 21 | HX174374 | 5' | 81 | + | Root |
| tae- miR2926 | osa-miR2926 | 73 | -15.20 | GGUCGUCGAC**A**UU**C**GGU**UG**U | 4 | 20 | HX031620 | 3' | 55 | - | Root |
| tae-miR2927 | osa-miR2927 | 69 | -19.10 | U**C**UCGUCG**CU**GAUGGAGCCCAUG | 3 | 23 | CD877524 | 3' | 61 | - | Root |
| tae-miR3476 | gra-miR3476 | 62 | -11.80 | **AGU**GACUGGAUUUGUUGACAA | 3 | 21 | CA691282 | 5' | 38 | - | Leaf |
| tae-miR3513 | ahy-miR3513 | 51 | -12.20 | U**G**AA**C**UUCU**C**A**U**UUUGUCAUC | 4 | 21 | HX073043 | 5' | 33 | + | Leaf |
| tae-miR3522 | gma-miR3522 | 129 | -35.75 | **U**GACC**UU**AUGAGCAGCUGA | 3 | 19 | CJ775590 | 3' | 47 | - | Root |
| tae-miR3627 | vvi-miR3627 | 87 | -48.60 | UUGUCGCAGGAGAGACG**C**CAC**G** | 2 | 22 | GH731337 | 5' | 64 | - | Seed |
| tae-miR3633 | vvi-miR3633b | 188 | -62.10 | **U**UUCCC**G**AUGCC**UC**CCAUUCCUA | 4 | 23 | CJ796550 | 5' | 52 | - | Root |
| tae-miR3635 | vvi-miR3635 | 57 | -13.00 | AU**G**AUGUCCCA**G**ACAUGCCUC | 2 | 21 | CD884271 | 5' | 52 | - | Leaf |
| tae-miR3636 | vvi-miR3636 | 72 | -17.30 | UCGGUUUGCUUC**C**UUGA**-**AGAUU**G** | 3 | 24 | CK156910 | 3' | 43 | - | Crown |
| tae-miR3954 | csi-miR3954 | 91 | -40.40 | -G**C**ACA**C**AGAAAUCACGGUCA | 3 | 20 | CD871085 | 3' | 50 | - | Root |
| tae-miR4364 | gma-miR4364b | 77 | -25.70 | UAACAACAGCGG**C**AG---CUUCUU | 3 | 21 | CJ529868 | 3' | 48 | + | Seed |
| tae-miR4367 | gma-miR4367 | 75 | -14.20 | AGCAUCAUAUC**A**CCU**U**CAU**U**G | 3 | 21 | CA486336 | 3' | 38 | - | Anther |
| tae-miR4411 | gma-miR4411 | 122 | -20.73 | **C**UAU**AA**UAACUAAUUUGUCGG**A** | 4 | 22 | HX198237 | 3' | 27 | - | Root |
| tae-miR4993 | gma-miR4993 | 72 | -36.30 | GAGCGGCGGCGG**C**GG**C**GGA**G**G | 3 | 21 | JZ885047 | 5' | 90 | + | Seedling |
| tae-miR4995 | gma-miR4995 | 86 | -33.83 | AGGCAGUGGCUUGGUUAAG**GG** | 2 | 21 | BE401706 | 5' | 57 | + | Leaf |
| tae-miR5017 | ath-miR5017 | 130 | -25.54 | AUUUGUUACU**U**A**G**U**A**GGAA**N**G | 4 | 21 | CA685734 | 3' | 29 | + | Leaf |
| tae-miR5034 | gma-miR5034 | 132 | -45.00 | GGUACC--UUCAGAUAGUCU**G**A | 3 | 20 | CA487066 | 5' | 45 | - | Anther |
| tae-miR5039 | gma-miR5039 | 236 | -35.01 | CCCUUUUUU**U**A**A**C**U**UUGCAUG | 3 | 21 | BJ287990 | 3' | 33 | - | Seed |
| tae-miR5040 | gma-miR5040 | 245 | -50.03 | **U**UGAUAUAUAACAAGCAU**A**AG | 2 | 21 | HX080066 | 5' | 24 | + | Leaf |
| tae-miR5056 | bdi-miR5056 | 104 | -12.24 | AGGAAGAACCGGUAAUAAGCA | 0 | 21 | CA620777 | 5' | 43 | + | Leaf |
| tae-miR5059 | bdi-miR5059 | 52 | -22.00 | CGG**G**C**G**GGGCAGCACCACC**G** | 3 | 20 | CA625260 | 5' | 85 | - | Leaf |
| tae-miR5063 | bdi-miR5063 | 79 | -28.20 | UCCACUG**AG**AAAGGCU**G**UUGCU | 3 | 22 | BJ261708 | 3' | 50 | + | Spike |
| tae-miR5064 | bdi-miR5064 | 96 | -54.30 | **U**GAAUUUGUCCAUAGCAUCA**U** | 2 | 21 | FL488864 | 5' | 33 | + | Anther |
| tae-miR5070 | bdi-miR5070 | 117 | -36.40 | AAC**UA**UAAGUAGG**A**UCA**A**AGG | 4 | 21 | HX019013 | 3' | 33 | + | Root |
| tae-miR5075 | osa-miR5075 | 131 | -54.93 | UUCUCC**C**U**G**GCC**U**CCGUCCGC | 3 | 21 | HX127214 | 3' | 71 | - | Root |
| tae-miR5076 | osa-miR5076 | 66 | -17.00 | **U**AAAUGGGAGCAGAGCAGGUUU | 1 | 22 | CA681413 | 3' | 45 | - | Leaf |
| tae-miR5082 | osa-miR5082 | 133 | -131.10 | **C**GCGAUGAUGGCCGCGCGGG**C**UCA | 2 | 24 | CV763765 | 3' | 75 | + | Crown |
| tae-miR5083 | osa-miR5083 | 80 | -14.00 | AGACUACAAUUAUCUGAUCA | 0 | 20 | CJ684590 | 3' | 30 | + | Anther |
| tae-miR5161 | osa-miR5161 | 165 | -49.98 | U**U**UGGA**A**CAGAGGGAGUAUA | 2 | 20 | FR854792 | 3' | 40 | - | Egg Cell |
| tae-miR5167a | bdi-miR5167 | 84 | -28.30 | CCACUUUGGGUGUCAUUGGU**G** | 1 | 21 | BQ609243 | 3' | 52 | + | Endosperm |
| tae-miR5167b | bdi-miR5167 | 91 | -22.70 | CCAAUGACACCCA**A**AGUGG**CG** | 3 | 21 | BQ609243 | 5' | 57 | - | Endosperm |
| tae-miR5169a | bdi-miR5169 | 149 | -42.50 | GUUUGACCAA**C**U**C**U**A**UAGAA**A** | 4 | 21 | CJ697097 | 5' | 33 | - | Shoot |
| tae-miR5169b | bdi-miR5169 | 139 | -37.00 | UUUGACCAAG--U**C**UAGAA**A**A | 4 | 19 | CJ697097 | 5' | 32 | + | Shoot |
| tae-miR5171 | bdi-miR5171 | 87 | -18.80 | ACUUA**U**U**U**UGGGACGGAAG**G**A | 3 | 21 | CJ796306 | 5' | 43 | + | Root |
| tae-miR5174a | bdi-miR5174 | 86 | -56.50 | CUCCGUUCCA**A**AA**U**AGAU**-**G**A**C | 4 | 21 | CJ901160 | 5' | 43 | - | Seedling |
| tae-miR5174b | bdi-miR5174b | 84 | -65.80 | CA**U**C**UA**UU**C**UGGAACGGAGGG | 4 | 21 | CD927329 | 3' | 52 | + | Seed |
| tae-miR5183 | bdi-miR5183 | 91 | -39.30 | UAUUUGGACAAAUUU**A**AG**A**CA | 2 | 21 | HX182761 | 3' | 24 | - | Root |
| tae-miR5203 | bdi-miR5203 | 57 | -26.20 | ACUUAUUAUGGA**U**CGGAGGGA | 1 | 21 | CB307280 | 3' | 43 | - | Stems |
| tae-miR5225 | mtr-miR5225b | 179 | -59.27 | UCGCAGGAGAGAUGACACC**GAC** | 3 | 22 | CJ773534 | 5' | 59 | - | Root |
| tae-miR5233 | mtr-miR5233 | 90 | -33.50 | GAGGAGGAU-GCCGUCUGG**CU** | 3 | 20 | AL815334 | 5' | 65 | + | Embryo |
| tae-miR5234 | mtr-miR5234 | 146 | -31.72 | UUUUGUUG--GAUGGCAGAAG | 2 | 19 | CA709300 | 3' | 42 | - | Kernel |
| tae-miR5254 | mtr-miR5254 | 171 | -40.61 | A**A**GAGGUGGAAGCAUUU**C**UG**U** | 3 | 21 | JZ890365 | 3' | 43 | + | Seedling |
| tae-miR5265 | mtr-miR5265 | 92 | -29.00 | **CAC**UGAUGUUGGAAUGGU**C**A | 4 | 20 | CA724348 | 3' | 45 | + | Seedling |
| tae-miR5272 | mtr-miR5272e | 96 | -27.50 | GAAUUGA**A**UUAUG**C**U**C**GGAUA**G**AC | 4 | 24 | CJ579659 | 3' | 38 | - | Anther |
| tae-miR5288 | mtr-miR5288 | 54 | -18.40 | C**G**GCAUUGAAGAACAU**U**GGGAUCA | 2 | 24 | JZ883198 | 3' | 46 | + | Seedling |
| tae-miR5291 | mtr-miR5291a | 142 | -43.03 | G**A**UUGAUGGAUGGA-UGGAUGGAU | 2 | 23 | CJ872025 | 3' | 43 | + | Root |
| tae-miR5298 | mtr-miR5298a | 240 | -60.35 | **A**GGAU**G**AUGAU**G**AUGAAGAUGAAG | 3 | 24 | HX010477 | 5' | 38 | - | Root |
| tae-miR5338 | osa-miR5338 | 101 | -38.20 | UGAAGCUUCAGUUG**A**UUG**C**UAU | 2 | 22 | HX167760 | 3' | 36 | + | Root |
| tae-miR5386 | sbi-miR5386 | 97 | -45.80 | CGUCGCUGUCGCGCGCG**GC**G | 2 | 20 | CJ623870 | 3' | 85 | + | cultured |
| tae-miR5387 | sbi-miR5387a | 453 | -149.25 | U**GG**CACGAACCGGUG**A**UAAAGG**U**UC | 4 | 25 | CD875071 | 5' | 52 | - | Leaf |
| tae-miR5490 | osa-miR5490 | 128 | -26.03 | UUGG**U**UU**A**U**A**UAUUU**U**GGACGG | 4 | 22 | CJ609733 | 5' | 32 | - | Shoot |
| tae-miR5502 | osa-miR5502 | 41 | -10.90 | UACG**U**AU-CGGAUACGCGAUAC | 2 | 22 | HX101882 | 5' | 48 | + | Leaf |
| tae-miR5508 | osa-miR5508 | 62 | -14.20 | UAGAUGG**U**CUG--CUGGUGUG**C** | 4 | 20 | GH728333 | 3' | 55 | - | Seed |
| tae-miR5523 | osa-miR5523 | 68 | -22.00 | UG**G**GGAGGAACAUAUUUACUAG | 1 | 22 | HX094480 | 3' | 41 | - | Leaf |
| tae-miR5527 | osa-miR5527 | 57 | -15.00 | U**G**UCAGCCAG**A**GCAGUAACAG | 2 | 21 | HX034092 | 5' | 52 | - | Root |
| tae-miR5538 | osa-miR5538 | 81 | -16.40 | ACUGAACUCAAUCACUUGCUGC | 0 | 22 | HX028290 | 5' | 45 | - | Shoot |
| tae-miR5539 | osa-miR5539 | 48 | -29.24 | AAGAAAACG**U**AUGCGCGUG**UAC** | 4 | 22 | BE400386 | 5' | 45 | - | Florets |
| tae-miR5543 | osa-miR5543 | 78 | -24.70 | UA**-**GAAUG**A**UAUAUUUUCUU**U** | 3 | 20 | BE489270 | 3' | 15 | + | Shoot |
| tae-miR5562 | mtr-miR5562 | 64 | -12.00 | U**U**UGGAGUCUUUUG**A**AUGAAG | 3 | 21 | CJ923104 | 5' | 33 | + | Seedling |
| tae-miR5564 | sbi-miR5564b | 83 | -24.13 | GCAAUUCGUCGAACA**C**CUUG | 1 | 20 | CD887914 | 5' | 50 | - | Seed |
| tae-miR5565a | sbi-miR5565a | 110 | -60.60 | AACACAUGUGGAUUGAGG**U**GAAUC | 1 | 24 | CA485136 | 3' | 42 | + | Anther |
| tae-miR5565b | sbi-miR5565a | 116 | -62.60 | AACAC**U**UGUGGAUUGA**U**GCGAAUC | 2 | 24 | CA485136 | 3' | 42 | - | Anther |
| tae-miR5565c | sbi-miR5565c | 98 | -59.80 | U**CCA**ACACAUGUGGAUUGAGGUG | 3 | 23 | CA485136 | 3' | 48 | + | Anther |
| tae-miR5565d | sbi-miR5565e | 139 | -79.70 | UUGUUUGGAUGUUGUCGGA | 0 | 19 | CD491292 | 5' | 42 | + | Seedling |
| tae-miR5565e | sbi-miR5565e | 141 | -78.30 | UUGUUUGGAUGU**A**GU**GA**GA | 3 | 19 | CD491292 | 5' | 37 | - | Seedling |
| tae-miR5565f | sbi-miR5565f | 122 | -72.10 | UAGUCGGAUU**CGC**AUCAAUC | 3 | 20 | CA485136 | 5' | 45 | + | Anther |
| tae-miR5565g | sbi-miR5565g | 105 | -46.40 | UUCA**UC**UCAAUCCACAU**G**U**A**UUGG | 4 | 24 | CD491292 | 5' | 38 | + | Seedling |
| tae-miR5565h | sbi-miR5565g | 115 | -55.00 | A**U**ACAUGUGGAUUGAGAUGAAU**C**C | 2 | 24 | CD491292 | 3' | 38 | - | Seedling |
| tae-miR5568a | sbi-miR5568c | 93 | -31.80 | UCUGUUCC**C**AA**A**U**A**UAAGUCG | 3 | 21 | CK162079 | 5' | 38 | - | Crown |
| tae-miR5568b | sbi-miR5568c | 109 | -26.48 | ACUUACA**U**UU**C**GGAACGGAGG | 2 | 21 | CD935109 | 3' | 48 | - | Ovary |
| tae-miR5568c | sbi-miR5568d | 51 | -9.70 | UG**UU**UUUU**G**UAGAU**U**CAUAGC | 4 | 21 | CA626241 | 5' | 29 | + | Leaf |
| tae-miR5568d | sbi-miR5568f | 53 | -14.20 | **A**UCUUAUA**U**UUUGGAAUGGAG | 2 | 21 | CD874459 | 5' | 29 | + | Leaf |
| tae-miR5568e | sbi-miR5568g | 75 | -33.40 | AGAACAUCUUAUAAUUUGGAA | 0 | 21 | BJ288879 | 3' | 24 | - | Seed |
| tae-miR5641 | ath-miR5641 | 84 | -22.83 | U**A**GAAGAAGAUG**A**AUAGAAUU**U** | 3 | 22 | CA646497 | 5' | 23 | - | Root |
| tae-miR5660 | ath-miR5660 | 45 | -11.30 | CAGG**A**GGUUAGUGCAAUGG**CA** | 3 | 21 | HX011631 | 3' | 52 | - | Root |
| tae-miR5679 | gma-miR5679 | 155 | -34.08 | UUGGUG-CCCAG**G**AGAAGUUGA | 2 | 21 | CK165790 | 5' | 52 | - | Crown |
| tae-miR5721 | bra-miR5721 | 197 | -14.11 | **AG**AAAUGGAG**A**GAGAAAUGGAGU | 3 | 23 | CJ593161 | 3' | 39 | - | Shoot |
| tae-miR5783 | gma-miR5783 | 71 | -26.50 | GA**G**G**G**CGACGGGGAGGACGCGC | 2 | 22 | CV771490 | 5' | 82 | - | Crown |
| tae-miR5806 | osa-miR5806 | 96 | -23.40 | A**G**CAGGCAAAGACAAUG**C**CGGC | 2 | 22 | HX179819 | 5' | 59 | - | Root |
| tae-miR5809 | osa-miR5809 | 65 | -30.90 | UCGUCGCCGGCGA**A**C**C**CAGC | 2 | 20 | CA623942 | 3' | 75 | - | Leaf |
| tae-miR5814 | osa-miR5814 | 77 | -21.10 | AAUCAAG**G**UAGGAA**U**C**U**UGCAAGU | 3 | 24 | CJ541433 | 5' | 38 | - | Seed |
| tae-miR5824 | osa-miR5824 | 60 | -12.10 | A**C**UCUGAU**C**AAGAAGUCAAUGGCG**A** | 3 | 25 | GH731296 | 3' | 44 | - | Seed |
| tae-miR5833 | osa-miR5833 | 74 | -35.50 | UCCUCCUCGGGCUC**C**UC**C**GGC | 2 | 21 | CA730826 | 5' | 76 | + | Pistils |
| tae-miR6026 | sly-miR6026 | 74 | -13.80 | U**A**UCUU**C**GC**A**AGAGUUGUAUUG**U** | 4 | 23 | CJ905157 | 5' | 35 | + | Seedling |
| tae-miR6034 | bna-miR6034 | 157 | -38.83 | UCUGAUGU**G**UAUAGCUUUGG**U** | 2 | 21 | BE497603 | 3' | 38 | + | Leaf |
| tae-miR6035 | bna-miR6035 | 51 | -11.00 | **A**GGAGUAGAAAAUGCAG**G**CGU | 2 | 21 | CJ688689 | 3' | 48 | - | Root |
| tae-miR6111 | cca-miR6111 | 53 | -15.90 | **C**CUUUAUGUCACGAUG**A**AUGA- | 3 | 21 | BE637850 | 5' | 38 | - | Spike |
| tae-miR6116 | cca-miR6116 | 72 | -14.30 | CAUGCUUGUG**U**UCAAA**A**GAU**A** | 3 | 21 | CJ710840 | 5' | 33 | - | Shoot |
| tae-miR6164 | nta-miR6164b | 123 | -71.70 | **G**UCA**AU**UAA**U**UUGAAACGGAG | 4 | 21 | CJ628344 | 3' | 33 | + | Callus |
| tae-miR6177 | hvu-miR6177 | 98 | -16.40 | UACCAUGGACAGAAGGCACUUA | 0 | 22 | HX074159 | 5' | 45 | - | Leaf |
| tae-miR6179 | hvu-miR6179 | 77 | -25.90 | AACCAGUCGA**A**GCCAGGGGGUU | 1 | 22 | CJ849289 | 5' | 59 | + | Root |
| tae-miR6180 | hvu-miR6180 | 92 | -32.78 | AGGGUGGAAGAAA**C**AGGGCG | 1 | 20 | CJ666586 | 5' | 60 | + | Shoot |
| tae-miR6181 | hvu-miR6181 | 66 | -30.00 | UGCUCUUCAUGGACUGCGGCGC**G** | 1 | 23 | CV759725 | 5' | 65 | + | Crown |
| tae-miR6182 | hvu-miR6182 | 61 | -18.40 | UGAGUGUGUGAUGGAUGGCUUU | 0 | 22 | CA619801 | 5' | 45 | + | Leaf |
| tae-miR6183 | hvu-miR6183 | 54 | -23.70 | UGAGCGAGUUGGCUGCAAGUUC | 0 | 22 | CJ687605 | 5' | 54 | + | Root |
| tae-miR6184 | hvu-miR6184 | 47 | -26.20 | C**C**GCGUCGGAUCUGG**A**CG**CG**C | 4 | 21 | DR735544 | 5' | 76 | + | Root |
| tae-miR6187 | hvu-miR6187 | 48 | -30.10 | UGAACAGGUUCGGCGACCUCA | 0 | 21 | CK204266 | 5' | 57 | + | Root |
| tae-miR6188 | hvu-miR6188 | 84 | -30.60 | GGUGGAUCGAUGAACCCGGCGA | 0 | 22 | HX172858 | 3' | 64 | - | Root |
| tae-miR6189 | hvu-miR6189 | 148 | -48.05 | AGGUGAUGCUGUGGUGAUCU | 0 | 20 | HX071645 | 5' | 50 | + | Leaf |
| tae-miR6190 | hvu-miR6190 | 96 | -36.80 | CGAGGAAAGGAAGAAGCCAUG | 0 | 21 | HX028627 | 5' | 52 | - | Shoot |
| tae-miR6191a | hvu-miR6191 | 71 | -46.50 | UAGAUUUGUCUAGAUAUG**G**A | 1 | 20 | CA662760 | 5' | 30 | + | Leaf |
| tae-miR6191b | hvu-miR6191 | 77 | -43.20 | UAGAUUUGUCUA**C**AUA**C**G**G**A | 3 | 20 | CA662760 | 5' | 35 | - | Leaf |
| tae-miR6192 | hvu-miR6192 | 53 | -21.30 | **G**AGGAGAGG**AA**GGA**G**GGGAUCU | 4 | 22 | CJ688412 | 5' | 59 | + | Root |
| tae-miR6193 | hvu-miR6193 | 176 | -62.78 | CUCUGCCACCGGUCCAUGACGAC | 0 | 23 | HX022800 | 5' | 65 | - | Shoot |
| tae-miR6195 | hvu-miR6195 | 111 | -53.50 | **C**GAGUACGU**G**GUAGGGAUGAG | 2 | 21 | HX125960 | 3' | 57 | + | Root |
| tae-miR6196 | hvu-miR6196 | 123 | -53.50 | AGGACGAGGAGAUGGAGAGGA | 0 | 21 | BJ312790 | 5' | 57 | + | Spike |
| tae-miR6199 | hvu-miR6199 | 127 | -39.70 | CCACAGAAUUCUCACAGUGA**C**GG | 1 | 23 | CJ925001 | 5' | 52 | + | Seedling |
| tae-miR6202 | hvu-miR6202 | 71 | -13.10 | UGAAG**C**UUUUAAGCA**C**UGAA | 2 | 20 | CK206121 | 5' | 35 | + | Crown |
| tae-miR6204 | hvu-miR6204 | 55 | -15.90 | AGGAGAAUAAUUAGAGCUGUGA | 0 | 22 | AL812079 | 3' | 36 | + | Endosperm |
| tae-miR6205 | hvu-miR6205 | 72 | -22.10 | AG**C**AUG**C**UUGGAUACGUUUUAGU | 2 | 23 | CK162343 | 5' | 39 | - | Crown |
| tae-miR6207 | hvu-miR6207 | 69 | -33.80 | UGGACGACCUGGGCGCCGACG | 0 | 21 | HX139346 | 3' | 76 | - | Root |
| tae-miR6209 | hvu-miR6209 | 66 | -19.60 | **CC**AGAUCAG**G**AAGA**A**GUGCG | 4 | 20 | CJ692917 | 5' | 55 | + | Shoot |
| tae-miR6213 | hvu-miR6213 | 209 | -47.90 | ACAGAUU**A**CUACAGACUG**U**UC | 2 | 21 | CA646146 | 3' | 38 | - | Root |
| tae-miR6214 | hvu-miR6214 | 176 | -69.17 | CGACGACGACGAGCACGACA | 0 | 20 | CJ684451 | 3' | 65 | + | Anther |
| tae-miR6220 | sbi-miR6220 | 75 | -23.00 | CUCC**G**U**AUA**AAAUUAUAAGACAUU | 4 | 24 | HX083836 | 5' | 25 | - | Leaf |
| tae-miR6224a | sbi-miR6224 | 76 | -21.90 | CUUAUAU**UA**UAGGACGGAGGG | 2 | 21 | CK203800 | 3' | 43 | + | Root |
| tae-miR6224b | sbi-miR6224 | 76 | -25.30 | CUCCGUCCUA**UA**AUAUAAG**AU** | 4 | 21 | CK203800 | 5' | 33 | - | Root |
| tae-miR6225 | sbi-miR6225 | 127 | -20.35 | AACUAG**G**CUCAAAAGAUUC**G**UCU- | 3 | 23 | CA484899 | 3' | 39 | - | Anther |
| tae-miR6233 | sbi-miR6233 | 73 | -20.70 | CA**U**GU**G**UGGUUUUGGUAAUU**G**AUG | 3 | 24 | AL816409 | 3' | 38 | - | Embryo |
| tae-miR6246 | osa-miR6246 | 71 | -39.90 | **G**UGGGGAUUUCCUGCCGGAGG**G**A | 2 | 23 | CV774566 | 5' | 65 | + | Crown |
| tae-miR6248 | osa-miR6248 | 87 | -45.70 | UAUUU**C**A**A**GAUGGAGGUAGUA | 2 | 21 | HX159375 | 3' | 33 | - | Root |
| tae-miR6249 | osa-miR6249a | 60 | -35.40 | CG**A**G**U**A**C**GAGC**A**CGCCGGCGGC | 4 | 22 | HX114793 | 3' | 77 | - | Leaf |
| tae-miR6253 | osa-miR6253 | 60 | -21.10 | GAGGAAAGUGGGCAGUUGGG**C**U | 1 | 22 | CA719683 | 5' | 59 | + | Kernel |
| tae-miR6275 | ppe-miR6275 | 108 | -28.75 | **CU**UGGAAG**G**AGCAAGGGGAAGC | 3 | 22 | CV758983 | 3' | 59 | - | Crown |
| tae-miR6276 | ppe-miR6276 | 81 | -32.00 | **C**AAGG**GG**CAUACAAAUAUUC- | 4 | 20 | BJ218315 | 3' | 40 | - | Spike |
| tae-miR6283 | ppe-miR6283 | 364 | -93.11 | CAAA**G**GG**U**GAGUGGGAAAAU**G** | 3 | 21 | CV762462 | 3' | 48 | + | Crown |
| tae-miR6426 | ptc-miR6426a | 132 | -48.50 | G-GGAGAC**GG**UGGA**G**GUGAAGA | 4 | 21 | CK209824 | 5' | 62 | + | Crown |
| tae-miR7488 | ghr-miR7488 | 154 | -40.80 | **CCA**UGACUACAGGGGACAAAA | 4 | 21 | CA653159 | 5' | 48 | + | Root |
| tae-miR7494 | gra-miR7494c | 186 | -53.95 | AUG**C**AGGAAAACAGAGGGAGAA**CU** | 3 | 24 | GH725517 | 3' | 46 | + | Seed |
| tae-miR7497 | ghr-miR7497 | 201 | -46.57 | **GA**AUGUGGACUGUCA**A**AUGGGUU | 3 | 23 | CJ785528 | 5' | 43 | - | Root |
| tae-miR7512 | ghr-miR7512 | 63 | -12.50 | UGC**C**ACU**AA**UAGUUAUGCAUG | 3 | 21 | HX050906 | 3' | 38 | + | Leaf |
| tae-miR7714 | bdi-miR7714 | 59 | -46.30 | UA**C**UU**C**CUC**C**GAUC**C**AUAUUACUU | 4 | 24 | CK205260 | 5' | 38 | + | Crown |
| tae-miR7725 | bdi-miR7725b | 93 | -22.50 | AUGCU**U**CA**U**CUCA**G**AUUUGAC | 3 | 21 | CJ602894 | 3' | 38 | - | Shoot |
| tae-miR7730 | bdi-miR7730 | 82 | -28.00 | A**G**CUUUCUCCCGCAGCUGUUC-GU | 2 | 23 | CK167575 | 5' | 57 | + | Crown |
| tae-miR7733 | bdi-miR7733 | 160 | -64.45 | **U**CUGCGUUGGCGA**C**GGCGAGAAGC | 2 | 24 | BJ267448 | 3' | 67 | - | Pistil |
| tae-miR7735 | bdi-miR7735 | 52 | -17.30 | CCGGU**GU**GAG**A**CAAGAGACGCGGC | 3 | 24 | BE424399 | 5' | 67 | - | Endosperm |
| tae-miR7748 | bdi-miR7748a | 74 | -8.80 | **U**AUAUGUUUUCU**U**UUGUUGG**G**C**C**G | 4 | 24 | HX141463 | 5' | 38 | + | Root |
| tae-miR7749 | bdi-miR7749 | 135 | -64.40 | AUCGUCGAGGGCGGAG**GCG**G**A**GGC | 4 | 24 | HX200059 | 3' | 75 | + | Root |
| tae-miR7768a | bdi-miR7768a | 162 | -77.78 | **U**CCGGU**A**CGA**A**GACGGCCCCG | 3 | 21 | CK195269 | 5' | 71 | - | Root |
| tae-miR7768b | bdi-miR7768a | 124 | -65.10 | CGGCG**G**CGUCCUCGA**G**CGGGAG | 2 | 22 | CA721482 | 5' | 82 | - | Kernel |
| tae-miR7773 | bdi-miR7773 | 183 | -78.53 | UUUU**CUG**UUCGGCUGACACG**A** | 4 | 21 | BJ261916 | 3' | 48 | - | Spike |
| tae-miR7775a | bdi-miR7775 | 114 | -47.80 | ACCG**AG**UU**U**UUCUGAAGCAC**U**AGU | 4 | 24 | CJ801823 | 5' | 42 | - | Root |
| tae-miR7775b | bdi-miR7775 | 112 | -55.70 | CUAGUGCUU**C**AGA-AAAAC**U**CGGUU | 3 | 24 | CJ801823 | 3' | 40 | + | Root |
| tae-miR7777 | bdi-miR7777 | 112 | -30.10 | UGAGAUGGUGU**A**U**U**UUG**UC**GG | 4 | 21 | CJ569196 | 3' | 43 | - | Seed |
| tae-miR7782 | bdi-miR7782 | 89 | -30.30 | ACCUGCUCUGAU**G**CCAUGUUG**G**GA | 2 | 24 | HX152726 | 3' | 54 | + | Root |
| tae-miR7786 | bdi-miR7786 | 158 | -43.03 | UGCACAAACUGUGG**G**GU**G**GU**U**GG**A** | 4 | 24 | GH731786 | 5' | 54 | - | Ovary |
| tae-miR7814 | ptc-miR7814 | 54 | -9.80 | U**U**GAUUGUUUUUA**A**GCUUUGA | 2 | 21 | HX089340 | 5' | 24 | + | Leaf |
| tae-miR7828 | ptc-miR7828 | 77 | -19.90 | **CC**UGACAUGGACAC**U**AAAAUC | 3 | 21 | DR736455 | 3' | 43 | + | Crown |
| tae-miR7829 | ptc-miR7829 | 130 | -39.55 | **G**C**U**CAGAA**C**C**A**CCAAGCCCAC | 4 | 21 | CJ729435 | 5' | 62 | + | cultured |
| tae-miR8014 | stu-miR8014 | 72 | -21.80 | AUUGU**A**U**UG**UAUUGUAUUGUAUU**G** | 4 | 24 | CJ578962 | 3' | 21 | - | Anther |
| tae-miR8015 | stu-miR8015 | 57 | -16.30 | **A**UUU**U**AUUUUCAAGGUCCAAUA**AU** | 4 | 24 | CV762614 | 3' | 21 | - | Crown |
| tae-miR8044a | stu-miR8044 | 96 | -25.40 | UCUCC**U**GC**C**AUAUUUGAAAC**A** | 3 | 21 | DR739392 | 3' | 38 | - | Crown |
| tae-miR8044b | stu-miR8044 | 146 | -38.30 | UUUCAAAUAUGG**CA**GGAGAUG | 2 | 21 | DR739392 | 5' | 38 | + | Crown |
| tae-miR8123 | ppe-miR8123 | 183 | -65.90 | **C**UGUGCCAU**C**GC**G**CAAGC | 3 | 18 | CA620960 | 3' | 67 | + | Leaf |
| tae-miR8135 | cpa-miR8135 | 37 | -8.80 | **UGA**AUUUUGCAGGGUUGAU | 3 | 19 | HX082574 | 5' | 37 | + | Leaf |
| tae-miR8154 | cpa-miR8154 | 65 | -21.50 | GAGAGGAGGAGAUGAAGAGG**AU** | 3 | 22 | CB307389 | 5' | 50 | + | Stems |
| tae-miR8595 | atr-miR8595 | 198 | -70.10 | C**U**C**C**A**C**UUGAAGGUCUUGGCC | 3 | 21 | BE500182 | 3' | 57 | - | Spike |
| tae-miR8659 | gra-miR8659a | 74 | -13.40 | **G**AUUU**G**UUAAGGUUGUU**G**GUGG**G**A | 4 | 24 | CF133375 | 5' | 42 | + | Anthers |
| tae-miR8728 | gra-miR8728 | 174 | -85.30 | CGGGCUUGGGC**UC**AA**GC**UUAGGCU | 4 | 24 | GH722898 | 3' | 63 | + | Leaf |
| tae-miR9555 | bra-miR9555a | 140 | -43.60 | **AUU**UAAGCUUUACGGGAAACC | 3 | 21 | CK168001 | 5' | 38 | - | Crown |
| tae-miR9557 | bra-miR9557 | 79 | -20.40 | UUUUGCGUUUCAA**A**UCGGUC**A** | 2 | 21 | HX136407 | 5' | 38 | - | Root |
| tae-miR9567 | bra-miR9567 | 99 | -23.70 | AAACUAU**C**UGU**C**UUGCU**A**AGA | 3 | 21 | CJ550024 | 3' | 33 | - | Anther |
